# Targeting Hsp70 triggers ferroptosis: a novel anti-cancer mechanism of a marine natural product in prostate cancer

**DOI:** 10.1007/s13659-025-00586-9

**Published:** 2026-02-05

**Authors:** Qiuyu Liu, Mengjing Cong, Chenghai Gao, Yonghong Liu, Junfeng Wang, Xueni Wang

**Affiliations:** 1 Guangxi Engineering Research Center for High-Value Utilization of Guangxi-Produced Authentic Medicinal Herbs, Institute of Traditional Chinese and Zhuang-Yao Ethnic Medicine, Guangxi University of Chinese Medicine, Nanning, 530200 China; 2 State Key Laboratory of Tropical Oceanography/Guangdong Key Laboratory of Marine Materia Medica, South China Sea Institute of Oceanology, Chinese Academy of Sciences, Guangzhou, 510301 China; 3 Guangxi Key Laboratory of Marine Drugs, Institute of Marine Drugs, Guangxi University of Chinese Medicine, Nanning, 530200 China; 4 Guangxi Innovation Center of Zhuang Yao Medicine, Institute of Traditional Chinese and Zhuang-Yao Ethnic Medicine, Guangxi University of Chinese Medicine, Nanning, 530200 China

**Keywords:** Prostate cancer, Heat shock protein 70, Androgen receptor, Membrane-associated O-acyltransferase domain protein 2, Ferroptosis, Marine natural product

## Abstract

Prostate cancer (PCa) remains one of the most common malignant tumors among men worldwide, typically relying on the androgen receptor (AR) signaling pathway. Inducing ferroptosis, a novel form of iron-dependent cell death, represents a promising strategy; however, its regulation by AR signaling is complex. The molecular chaperone heat shock protein 70 (HSP70) is critical for AR stability and function, yet its role as a therapeutic target in this context is underexplored. The anti-proliferative effect of the compound nidurufin (Nid) was assessed across PCa cell lines using MTT, clonogenic, and 3D spheroid assays. Ferroptosis was evaluated by transmission electron microscopy, reactive oxygen species (ROS) detection, and lipid peroxidation analysis. Mechanistic insights were gained through Western blot, qPCR, immunofluorescence, ChIP-qPCR, molecular docking, and cellular thermal shift assay (CETSA). In vivo efficacy was validated in a zebrafish xenograft model. Nid exhibited potent, selective anti-proliferative activity against AR-positive PCa cells, particularly 22Rv1 (IC₅₀ = 10.30 μM), and induced ferroptosis characterized by mitochondrial shrinkage and ROS accumulation. Mechanistically, Nid did not bind to AR, but it directly bound to HSP70, disrupting its chaperone function and leading to AR protein destabilization and transcriptional downregulation. This consequently suppressed the expression of the AR-target gene membrane-associated O-acyltransferase domain protein 2 (MBOAT2), a key ferroptosis suppressor enzyme. ChIP-qPCR confirmed AR directly binds the MBOAT2 promoter, and Nid treatment reduced this enrichment. In vivo, Nid significantly inhibited tumor growth and metastasis in a zebrafish xenograft model. Our study identifies Nid as a novel HSP70-targeted compound that triggers ferroptosis by disrupting the HSP70-AR-MBOAT2 axis. This work not only reveals a previously unrecognized connection between protein chaperone function and ferroptotic susceptibility but also positions HSP70 as a compelling therapeutic target for overcoming AR-pathway dependency in PCa.

**Graphical Abstract:**

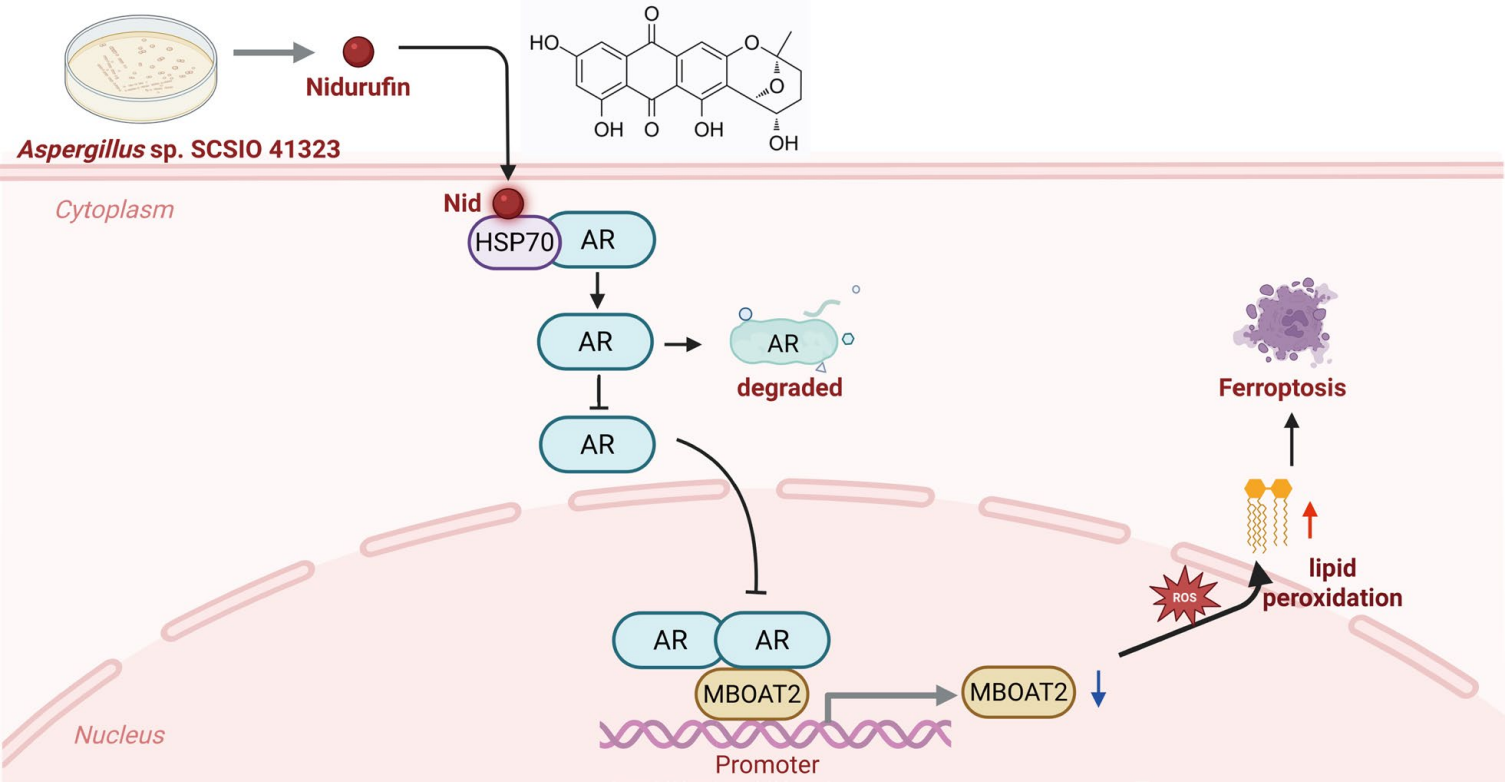

**Supplementary Information:**

The online version contains supplementary material available at 10.1007/s13659-025-00586-9.

## Introduction

In recent years, ferroptosis has emerged as a novel iron-dependent form of regulatory cell death, demonstrating significant potential in the field of cancer therapy [1, 2]. Distinct from apoptosis, necrosis, and autophagy, ferroptosis is fundamentally characterized by the inhibition of glutathione peroxidase 4 (GPX4) activity [3, 4]. This leads to the inability to reduce lipid peroxides, causing their excessive accumulation and subsequent disruption of cell membrane structures [5]. The ferroptosis process is tightly regulated, with the phospholipid metabolism pathway playing a pivotal role. Research indicates that membrane-associated O-acyltransferase domain protein 2 (MBOAT2), as a key ferroptosis inhibitor, catalyzes the incorporation of monounsaturated fatty acids (MUFAs) into phospholipids. This reduces the content of polyunsaturated fatty acid phospholipids (PUFA-PLs) [6, 7] in membranes, which are prone to lipid peroxidation, thereby effectively suppressing ferroptosis occurrence [8]. Notably, genomic studies confirm MBOAT2 as a direct transcriptional target of androgen receptor (AR), establishing a novel molecular link between oncogenic AR signaling and cellular ferroptosis susceptibility [9]. This suggests that targeting the AR-MBOAT2 axis to induce ferroptosis may offer a novel therapeutic approach for AR-positive prostate cancer (PCa).

In the exploration of novel strategies targeting AR, the heat shock protein (HSP) network has drawn significant attention from researchers. Heat shock protein 70 (HSP70), as a key molecular chaperone, interacts with multiple client proteins through its substrate-binding domain, participating in protein folding, stabilization, and activation processes [10]. In prostate cancer cells, the conformational stability and transcriptional function of AR are highly dependent on its interaction with the HSP70 or HSP90 molecular chaperone complex. Specifically, HSP70 promotes the correct folding of AR during its early maturation phase and collaborates with HSP90 to maintain AR in a high-affinity ligand-binding conformation [11, 12]. Disrupting this chaperone system leads to reduced AR protein stability and impaired nuclear translocation, offering an alternative strategy for indirectly inhibiting AR signaling distinct from direct antagonists [13]. Although HSP90 inhibitors (e.g., 17-AAG) have demonstrated clinical efficacy, their hepatotoxicity and resistance issues limit widespread application [14]. In contrast, HSP70 remains an under-explored target in PCa therapy.

Currently, various natural compounds, synthetic small molecules, and novel peptides have been demonstrated to induce ferroptosis in prostate cancer cells through distinct molecular mechanisms, primarily by disrupting intracellular antioxidant defense systems. Based on their core targets, these compounds can be categorized as follows: First, compounds targeting GPX4, such as evodiamine, which promotes GPX4 degradation by downregulating the E3 ubiquitin ligase TRIM26 [15], and dihydrochelerythrine, which directly downregulates GPX4 protein expression [16]; Second, compounds inhibiting SLC7A11, a key component of the cystine/glutamate reverse transporter (System Xc^−^). For example, arbutin inhibits its activity [17], while palbociclib downregulates its expression via the TRIB3/SOX2 axis [18]. Additionally, the novel cyclic peptide CCDC719-13 can specifically degrade SLC7A11 protein via the ubiquitin–proteasome pathway [19]. Third, natural products exerting effects through multi-pathway synergism exist, such as sinularin inducing oxidative stress by modulating the androgen receptor signaling pathway [20], and berberine chloride promoting ferroptosis by regulating the ROS/USP47/BACH1/HMOX1 signaling axis [21]. Although most studies remain in the preclinical stage, ferroptosis induction strategies offer novel therapeutic directions for refractory prostate cancer. Future research should focus on enhancing target specificity and advancing clinical translation.

Marine natural products represent an inexhaustible treasure of structural novelty and potent biological activities, offering promising leads for pharmaceutical development. In recent years, the exploration of extreme marine environments has emerged as a new frontier for biodiscovery [22]. Among them, anthraquinones are an important class of naturally occurring pigments with significant anticancer potential, which often function by intercalating into DNA, inhibiting topoisomerase II, and inducing apoptosis in cancer cells [23]. This study isolated and purified compounds from the solid-state fermentation extract of *Aspergillus versicolor* SCSIO 41323, successfully obtaining the compound nidurufin (Nid). It first revealed the mechanism by which Nid inhibits prostate cancer cell growth by targeting HSP70 to induce ferroptosis. We found that Nid specifically binds to the substrate-binding domain of HSP70, disrupting the stability of the HSP70-AR complex. This leads to reduced AR protein synthesis [24], decreased expression levels, and impaired nuclear translocation of AR. The resulting suppression of AR transcriptional activity further downregulates its downstream target gene MBOAT2, disrupting cellular lipid homeostasis. This promotes the accumulation of polyunsaturated fatty acid phospholipids and lipid peroxidation, ultimately triggering ferroptosis. We validated this novel HSP70-AR-MBOAT2 signaling axis using in vitro cell models and zebrafish xenograft models. This study not only reveals the unique molecular mechanism of Nid but also highlights the translational potential of targeting HSP70 to regulate AR function and induce ferroptosis as a therapeutic strategy for PCa.

## Results

### Nidurufin effectively inhibits the proliferation of prostate cancer cells

**Fig. 1:**
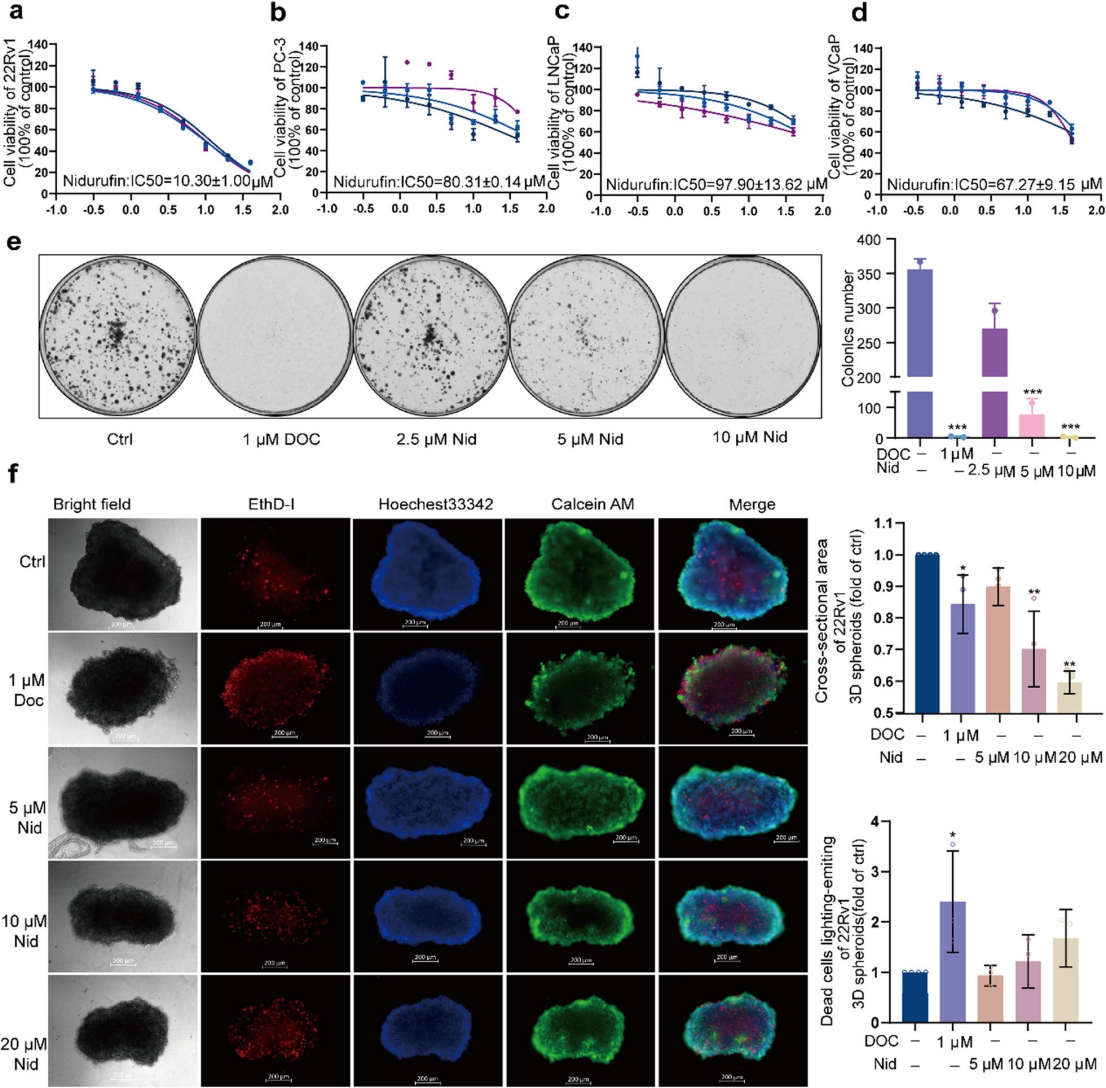
Effects of Nid on prostate cancer cell proliferation. **a**–**d** 22Rv1, PC3, LNCaP, VCaP cells were treated with different concentrations of Nid for 72 h, and the effect of compounds on cell survival was detected by MTT. **e** 22Rv1 cells were treated with different concentrations of Nid for 7 days. Crystal violet staining was used to detect the effect of the compounds on the formation of cell plate clones. **f** 22Rv1 cells were treated with different concentrations of Nid for 3 days, and the live/dead cell staining kit was used to detect the effect of the compound on the formation of 3D cell spheres. ^*^P < 0.05, ^**^P < 0.01 vs Ctrl

To systematically evaluate the anti-proliferative potential of Nid against PCa, we employed a comprehensive approach combining multiple cell viability and proliferation assays. The initial investigation aimed to determine the compound's potency across PCa cell lines with varying molecular characteristics. Using MTT assays, we performed dose–response profiling in four PCa cell lines: 22Rv1, VCaP, LNCaP, and PC3. While Nid demonstrated potent activity against 22Rv1 cells (IC₅₀ = 10.30 ± 1.00 μM) (Fig. 1a), the AR-negative PC3 cells exhibited intermediate sensitivity (IC_50_ = 80.31 ± 0.14 μM) (Fig. 1b), other AR-positive cell lines showed considerably reduced sensitivity, with IC₅₀ values of 97.90 ± 13.62 μM for LnCaP (Fig. 1c) and 67.27 ± 9.15 μM for VCaP (Fig. 1d). The results revealed a complex sensitivity pattern that could not be simply attributed to AR status. To further characterize the growth-inhibitory effects observed in 22Rv1 cells, we conducted additional functional assays. Colony formation assays demonstrated that Nid treatment significantly suppressed both the number and size of colonies in a dose-dependent manner (2.5, 5, and 10 μM) (Fig. 1e). The reduction in clonogenic capacity was particularly evident at the highest concentration tested, where colony formation was nearly completely abolished. Complementing these findings, 3D spheroid assays provided additional evidence of Nid's anti-proliferative activity. Treatment with Nid (5, 10, and 20 μM) for 72 h resulted in dose-dependent reductions in spheroid volume and integrity, accompanied by increased cell death compared to vehicle-treated controls (Fig. 1f). The 3D model effectively captured Nid's ability to disrupt tumor spheroid growth and maintenance. These collective findings establish that Nid possesses significant anti-proliferative activity against prostate cancer cells, with particularly pronounced effects on 22Rv1 cells. However, the heterogeneous response among AR-positive cell lines indicates that the compound's mechanism of action may involve additional molecular determinants beyond AR signaling. Further investigation is required to elucidate the specific factors underlying this selective sensitivity pattern.

### Nidurufin induces oxidative stress leading to ferroptosis in 22Rv1 cells

**Fig. 2:**
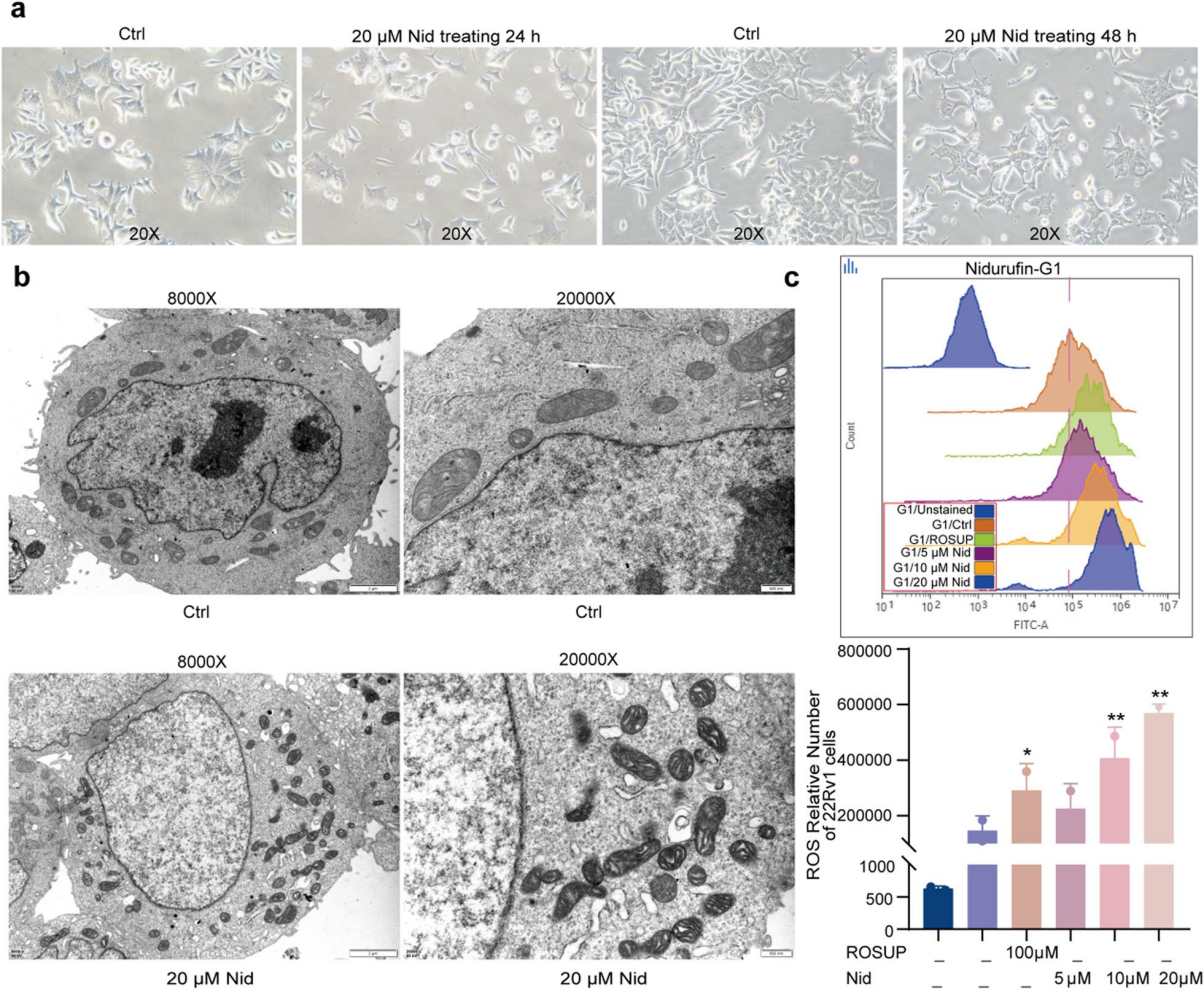
Effects of Nid on the morphology and ROS level in 22Rv1 cells. **a** 22Rv1 cells were treated with Nid for 24 and 48 h, and the cell morphology was shown under the light microscope. **b** 22Rv1 cells were treated with Nid for 48 h, and the internal structure of the cells was observed by transmission electron microscopy. **c** 22Rv1 cells were treated with Nid for 72 h, and the intracellular ROS level changes were detected by flow cytometry. ^*^P < 0.05, ^**^P < 0.01 vs Ctrl

To investigate the potential cell death mechanisms underlying Nid's anti-proliferative effects, we focused on 22Rv1 cells and employed morphological and biochemical approaches. Preliminary optical microscopy revealed a marked reduction in the density of Nid-treated 22Rv1 cells. Additionally, a portion of the cells exhibited significant morphological alterations, characterized by a transition from a “spreading” to a “round” morphology (Fig. 2a). To observe morphological changes in ultrastructure, we performed transmission electron microscopy (TEM) analysis after treating cells with 20 μM Nid for 48 h. The TEM images demonstrated classic features associated with ferroptosis, including mitochondrial atrophy, increased membrane density, reduced or absent cristae, and localized outer membrane ruptures (Fig. 2b). These morphological characteristics align well with established ferroptosis phenotypes described in recent literature [25, 26]. To quantitatively assess the oxidative stress component potentially driving ferroptosis [27], we measured intracellular ROS levels using flow cytometry. Treatment with Nid (5, 10, and 20 μM) for 72 h resulted in a significant, dose-dependent increase in ROS accumulation compared to vehicle-treated controls (Fig. 2c). The ROS levels in treated cells showed comparable induction to the positive control group treated with Rosup reagent (100 μM), confirming Nid's ability to induce substantial oxidative stress. The combination of morphological evidence from TEM and quantitative ROS detection provides compelling, though preliminary, evidence that Nid may induce ferroptosis in 22Rv1 cells. The observed mitochondrial ultrastructural changes, coupled with elevated ROS levels, represent key hallmarks of ferroptosis execution.

### Nidurufin induces oxidative stress by regulating the AR/MBOAT2 signaling pathway

**Fig. 3:**
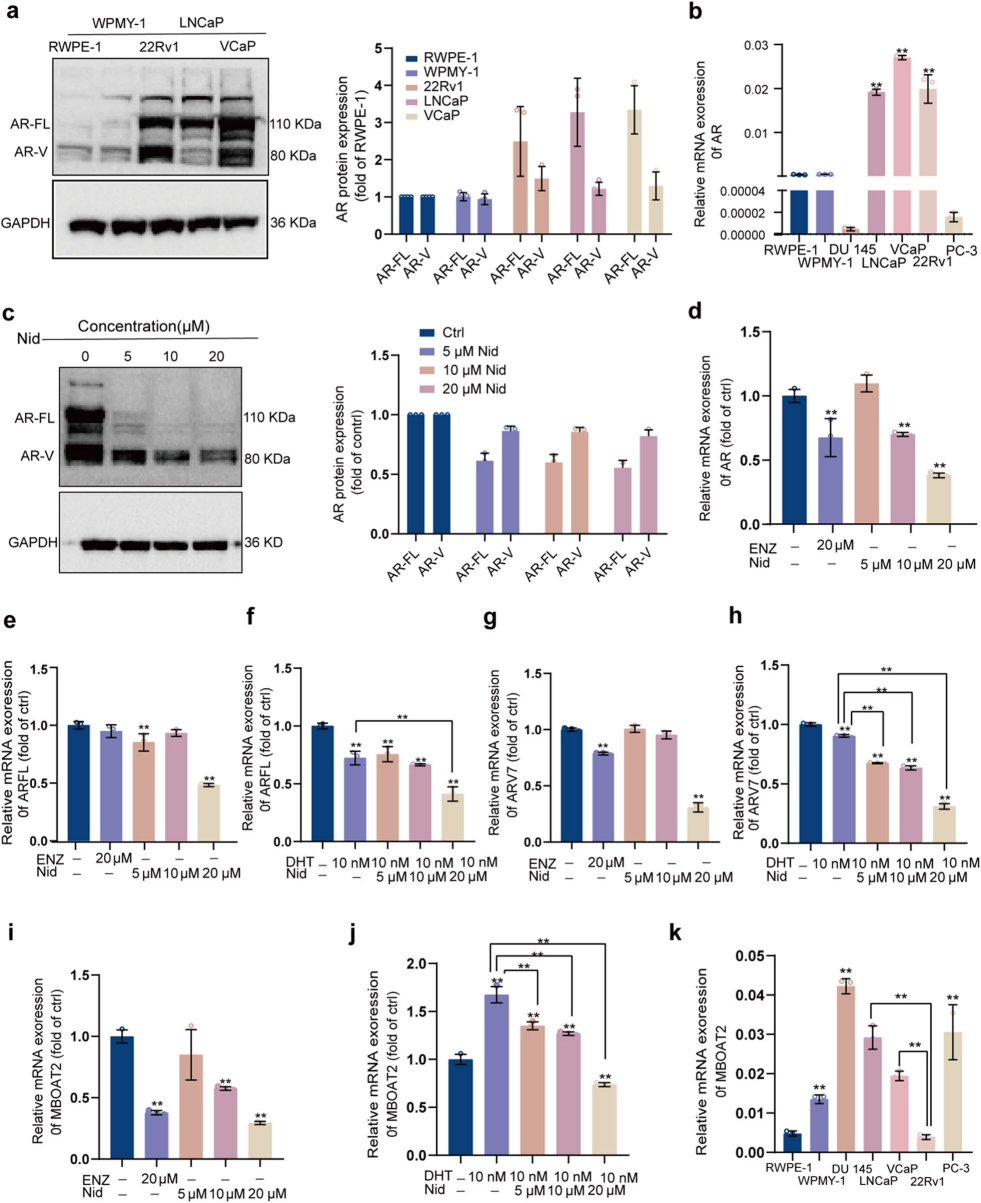
Nid's regulatory effect on the expression of AR and MBOAT2 molecules in 22Rv1 cells. **a** A comparative analysis of AR protein expression across different cell lines. **b** A comparative analysis of AR mRNA expression across different cell lines. **c** Changes in AR-FL, AR-V protein levels in 22Rv1 cells after treatment with different concentrations of Nid. **d** Changes in AR mRNA levels in 22Rv1 cells after treatment with different concentrations of Nid. **e** Changes in AR-FL mRNA levels in 22Rv1 cells after treatment with different concentrations of Nid. **f** Changes in AR-FL mRNA levels in 22Rv1 cells following treatment with varying concentrations of Nid with or without DHT. **g** Changes in AR-V mRNA levels in 22Rv1 cells after treatment with different concentrations of Nid. **h** Changes in AR-V mRNA levels in 22Rv1 cells following treatment with varying concentrations of Nid with or without DHT. **i** Changes in MBOAT2 mRNA levels in 22Rv1 cells after treatment with different concentrations of Nid. **j** Changes in MBOAT2 mRNA levels in 22Rv1 cells following treatment with varying concentrations of Nid with or without DHT. **k** qPCR analysis of MBOAT2 mRNA levels in different cell types. ^*^P < 0.05, ^**^P < 0.01 compared to the control group

Given that Nid exhibits the strongest inhibitory effect on 22Rv1 cells, which simultaneously express both the full-length and splice variant forms of the AR. To investigate how Nid disrupts redox homeostasis and triggers oxidative stress in 22Rv1 cells. We first analyzed the expression profiles of the full-length androgen receptor (AR-FL) and its splice variants (AR-V) across a series of prostate cell lines. Western blot and qPCR analyses confirmed that AR-FL and AR-V were highly expressed in 22Rv1 and VCaP cells, detected at extremely low levels in normal prostate cells (RWPE-1, WPMY-1), absent in AR-negative cells (DU145, PC3), and present only as AR-FL in LNCaP cells (Fig. 3a, b). Treatment of 22Rv1 cells with Nid resulted in a concentration-dependent decrease in both AR-FL protein (Fig. 3c) and mRNA (Fig. 3d) expression, with AR-FL showing a more pronounced inhibitory effect than AR-V. Previous studies indicate that phospholipid-modifying enzyme MBOAT2, as a ferroptosis suppressor, inhibits ferroptosis by reshaping phospholipid profiles [28]. Its transcription is positively regulated by AR, making it a key target gene in the AR signaling pathway of PCa. Combining AR antagonists with ferroptosis inducers significantly suppresses the growth of AR-positive PCa [29]. Given the role of AR in regulating lipid metabolism-related genes, we investigated whether Nid affects MBOAT2 expression. qPCR analysis revealed that high-concentration Nid significantly reduced AR-FL and AR-V mRNA levels, with an efficacy comparable to or stronger than that of the AR antagonist enzalutamide (ENZ) (Fig. 3e, g). Furthermore, Nid effectively blocked DHT-induced transcriptional activation of AR target genes. When DHT activated the AR signaling pathway, co-treatment with Nid concentration-dependently reversed this effect, reducing AR-FL and AR-V expression (Fig. 3f, h, Figure S1a) and significantly inhibiting MBOAT2 transcription (Fig. 3i, j). However, Nid did not significantly inhibit the expression of the AR target genes KLK3, TMPRSS2 and PCA3 (Figure S1). Multicellular line analysis confirmed that 22Rv1 cells exhibit the lowest basal MBOAT2 mRNA levels (Fig. 3k). As can be seen in Fig. 3a above, the expression levels of the AR-FL and its splice variants are comparable in 22Rv1 and VCaP cells, while the expression of MBOAT2 in 22Rv1 cells was significantly lower than that in VCaP cells (Fig. 3k). Among the three cell lines expressing the AR, we were surprised to find that MBOAT2 expression levels followed the order 22Rv1 < VCaP < LNCaP. Furthermore, the IC₅₀ values for Nid inhibition in these three cell lines also followed the same order: 22Rv1 < VCaP < LNCaP. These data suggest that the inhibitory effect of Nid on cell viability is negatively correlated with intracellular MBOAT2 expression levels. Therefore, we can infer that, when the expression of MBOAT2 in these cells is simultaneously affected, it is evident that 22Rv1 cells will suffer the most severe consequences. This data can elucidate why Nid exhibits the most pronounced effect specifically on 22Rv1 cells among these prostate cancer cells. These findings demonstrate that Nid's efficacy in AR-positive prostate cancer cells is mechanistically underpinned by its dual action of attenuating AR signaling and downregulating the ferroptosis suppressor MBOAT2.

### Nidurufin targets the HSP70/AR/MBOAT2 pathway to induce ferroptosis

**Fig. 4:**
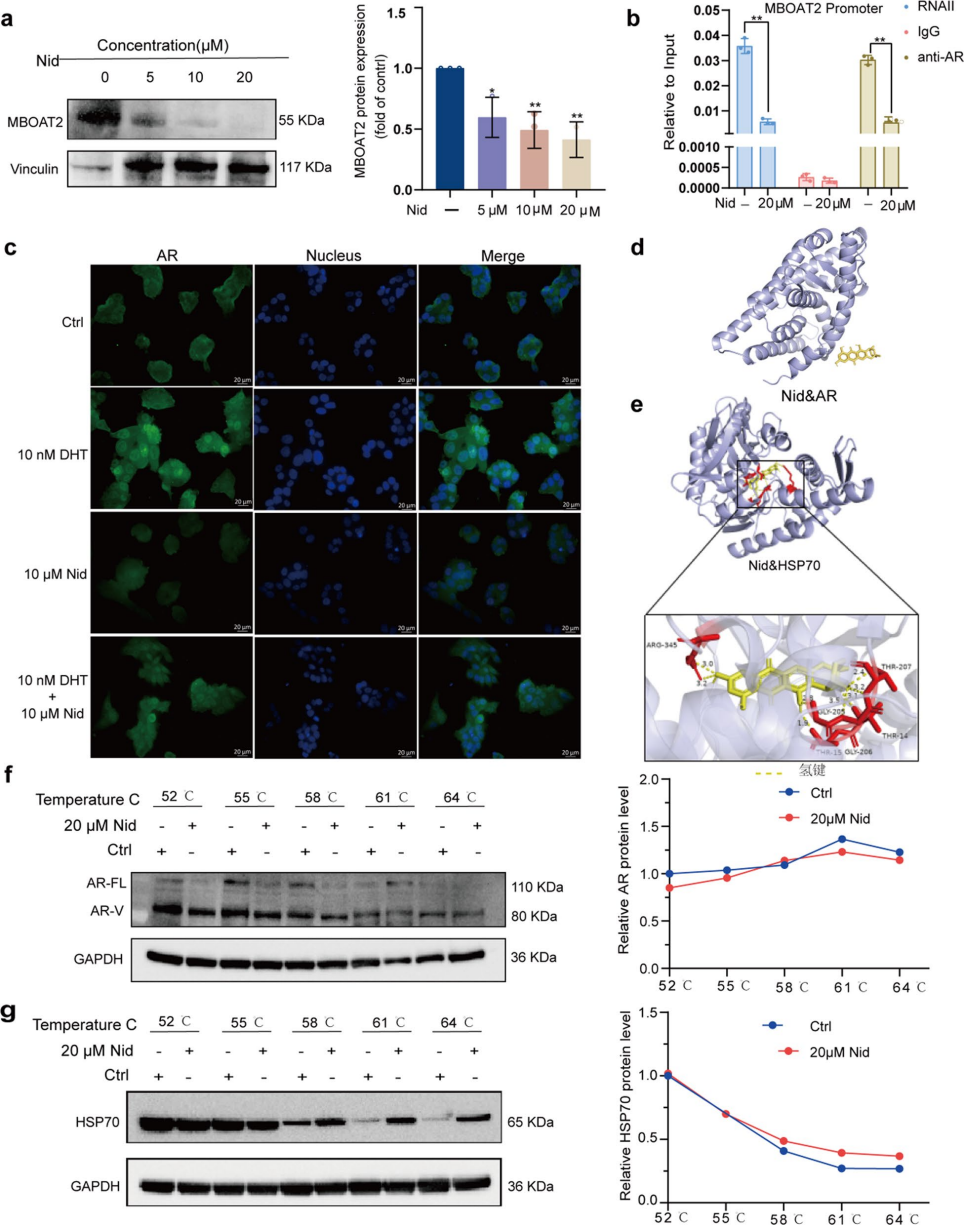
Nid induces ferroptosis by targeting the HSP70/AR/MBOAT2 axis. **a** Western blot analysis of MBOAT2 protein levels following Nid treatment at different concentrations. **b** ChIP-qPCR validation of direct AR binding to the MBOAT2 gene. **c** Immunofluorescence staining showing Nid's effect on AR protein expression in 22Rv1 cells. **d** Molecular interactions between Nid and AR proteins. **e** Molecular interactions between Nid and HSP70 proteins. **f** Cellular thermoshift assay demonstrating Nid's effect on AR thermal stability, including WB analysis and thermal shift profile. **g** Cellular thermoshift assay demonstrating Nid's effect on HSP70 thermal stability, including WB analysis and thermal shift profile. ^*^P< 0.05, ^**^P< 0.01 vs Ctrl

To further elucidate how Nid triggers oxidative stress in cells through the regulation of AR/MBOAT2, we conducted a series of experiments. Western blot analysis revealed a dose-dependent decrease in MBOAT2 protein expression following Nid treatment (Fig. 4a). Combined with the data presented in Fig. 3i, this indicated that Nid inhibited the transcriptional regulation of MBOAT2. Subsequently, we investigated whether Nid exerts a direct influence on AR's regulation of MBOAT2 transcription. ChIP-qPCR analysis confirmed that, under control conditions, there was significant enrichment of AR in the promoter region of MBOAT2, whereas Nid treatment significantly reduced this enrichment (Fig. 4b), suggesting that Nid diminished the direct transcriptional interaction between AR and MBOAT2. Furthermore, to validate the critical role of AR in Nid-induced ferroptosis, we examined the expression and localization of AR. Immunofluorescence staining demonstrated that the androgen receptor agonist DHT (10 nM) promoted nuclear accumulation of AR, while Nid (10 μM) significantly decreased AR protein levels and impeded its nuclear translocation (Fig. 4c). Notably, co-treatment of cells with DHT and Nid partially restored AR expression and localization, indicating that the effects of androgens could be reversed by Nid. Based on the aforementioned research findings, it is natural to speculate that Nid may exert a series of effects by directly binding to AR. However, to our surprise, both molecular docking analysis and cellular thermal shift assay ruled out the possibility of direct binding between Nid and AR (Fig. 4d, 4f), compelling us to explore alternative pathways. Considering the pivotal role of the heat shock protein family in maintaining AR stability, we hypothesize that Nid may indirectly influence the function of the AR signaling axis by interfering with the actions of heat shock proteins. Through molecular docking calculations, we assessed the molecular interactions between Nid and several heat shock proteins (Figure S2, Table S1) and discovered that Nid can form stable bonds with HSP70, primarily through hydrophobic interactions and hydrogen bonding (Tables 1, 2), specifically binding to distinct pockets within HSP70 (Fig. 4e). Subsequently, we employed cellular thermal shift assay to further validate the intracellular interaction between the two molecules. Under identical temperature conditions, cells treated with Nid exhibited significantly enhanced thermal stability of HSP70 and markedly reduced degradation (Fig. 4g), confirming the binding of Nid to HSP70. Collectively, these findings suggest that Nid disrupts the molecular chaperone function of HSP70 towards AR by binding to it, leading to AR protein instability and subsequent degradation. This results in a decreased of AR entering the nucleus, thereby inhibiting the transcription of MBOAT2. Consequently, the synthesis of the ferroptosis suppressor MBOAT2 protein is impaired, disrupting the redox balance and ultimately promoting lipid peroxidation and ferroptosis.

**Table 1:** Hydrophobic interactions between nidrurfin and HSP70 (PDB ID:3I33) protein

Index	Residue	AA	Distance	Ligand atom	Protein atom
1	38A	THR	3.67	2939	251

**Table 2:** Hydrophobic bonds between nidrurfin and HSP70 (PDB ID:3I33) protein

Index	Residue	AA	Distance H-A	Distance D-A	Donor Angle	Donor atom	Acceptor atom
1	14A	THR	2.31	3.12	138.78	61[Nam]	2918[O3]
2	15A	THR	1.93	2.70	134.58	2944[O3]	73[O3]
3	205A	GLY	2.10	2.81	126.79	1510[Nam]	2944[O3]
4	206A	GLY	2.58	3.52	160.68	1514[Nam]	2942[O3]
5	207A	THR	2.37	3.20	142.11	1518[Nam]	2942[O3]
6	207A	THR	2.87	3.78	156.93	2942[O3]	1521[O2]
7	275A	ARG	3.32	3.73	107.30	2081[Ng +]	2946[O3]
8	275A	ARG	3.54	3.93	106.07	2082[Ng +]	2946[O3]
9	345A	ARG	2.54	3.24	127.50	2623[Ng +]	2948[O3]
10	345A	ARG	2.18	2.96	135.43	2624[Ng +]	2948[O3]

### The effect of nidurufin on the development of prostate cancer in zebrafish xenografts

**Fig. 5:**
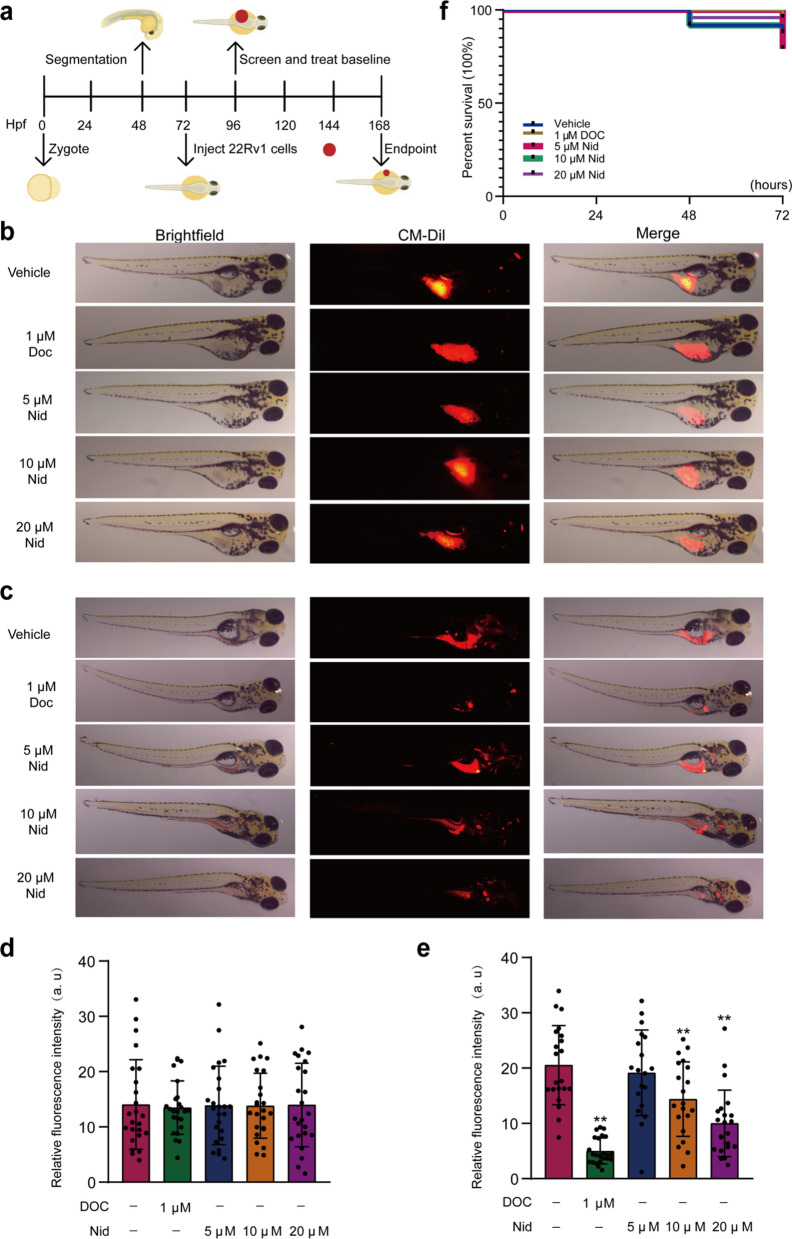
Nid inhibits the growth of xenografted prostate cancer in zebrafish. **a** Establishment of a zebrafish xenograft model. **b** Distribution of prostate 22Rv1 cells in zebrafish before drug administration after grouping. **c** Distribution of prostate 22Rv1 cells in zebrafish after 72 h of treatment with different concentrations of Nid. **d** Statistical distribution of 22Rv1 cells in zebrafish groups prior to drug administration. **e** Statistical distribution of 22Rv1 cells in zebrafish after 72 h of treatment with different concentrations of Nid. **f** Survival curve plotted from daily monitoring of zebrafish growth following Nid treatment. ^*^P < 0.05, ^**^P < 0.01 vs vehicle

While mouse models are a standard for in vivo validation, the zebrafish xenograft model is increasingly recognized as a powerful and complementary in vivo system for early-stage antitumor drug screening, especially when compound availability is limited. Recent work has effectively used zebrafish PDX models to reveal the critical role of the TRPM4 ion channel in PCa metastasis, demonstrating the model's value in discovering novel therapeutic targets [30]. Furthermore, studies have used zebrafish to evaluate PROTAC AR degraders specifically for PCa, showing a strong correlation between zebrafish data and compound efficacy [31–34]. These studies confirm that the zebrafish model is sufficiently convincing. To evaluate the in vivo antitumor efficacy of Nid, we employed a zebrafish xenograft model that provides a physiologically relevant system for assessing both primary tumor growth and metastatic behavior (Fig. 5a). This model was particularly suited for our study as it allows for real-time monitoring of tumor progression and drug effects in a living organism. Fluorescently labeled human 22Rv1 PCa cells were microinjected into the vitelline membrane gap of zebrafish embryos at post-fertilization day 3 (DP3). Prior to drug administration, the number of viable cells in zebrafish across all groups was comparable (Fig. 5b, d). Subsequently, embryos were treated with either Nid or the positive control drug Doc for 72 h. Tumor growth and spread were monitored and quantified daily via fluorescence microscopy. Compared to the solvent control group, Nid treatment exhibited a significant dose-dependent inhibition of tumor growth (Fig. 5c). Quantitative analysis revealed that at the highest concentration (20 μM), both fluorescence intensity and tumor area were significantly reduced (Fig. 5e), indicating the drug effectively suppresses proliferation and/or survival of cancer cells in vivo. Notably, Nid also effectively inhibited metastatic spread. Cancer cells in vehicle group frequently disseminated to the caudal region and other distal sites, whereas Nid-treated fish exhibited significantly reduced metastasis incidence and extent. During the experiment, a small number of zebrafish died in each group; however, there were no statistically significant differences in survival curves. This suggests that Nid exhibits no significant toxicity to zebrafish at the administered doses in this study (Fig. 5f). These in vivo results confirm that Nid effectively suppresses both local growth and metastatic spread of PCa xenografts.

## Discussion

Ferroptosis, as an emerging form of cell death, demonstrates significant potential in targeting drug-resistant tumors [35]. It is characterized by marked lipid peroxidation, ROS bursts [36], and distinctive mitochondrial ultrastructural alterations [37]. Our study reveals that Nid-treated prostate cancer cells exhibit typical ferroptosis ultrastructural features under transmission electron microscopy, including mitochondrial fission, increased membrane density, and reduced cristae structures. Flow cytometry confirmed that Nid induces ROS bursts in a concentration-dependent manner. Collectively, these findings establish ferroptosis as the primary mechanism of Nid-induced cell death. This discovery aligns strongly with recent perspectives suggesting that inducing ferroptosis may represent a breakthrough therapeutic strategy for prostate cancer [38].

Through mechanistic studies, we progressively elucidated the molecular targets and signaling pathways of Nid. Notably, Nid does not directly bind to AR but instead targets its key molecular chaperone, HSP70. Molecular docking predicted precise binding patterns between Nid and HSP70, while CETSA experiments confirmed at the cellular level that this binding significantly enhances HSP70 thermal stability. HSP70 plays a crucial role in maintaining the correct conformation and stability of unliganded AR, forming complexes with proteins such as HSP90 and Hop to jointly assist AR folding and activation [39]. We speculate that Nid binding to HSP70 may occupy its substrate-binding domain or induce conformational changes, disrupting its normal chaperone cycle and thereby increasing AR susceptibility to degradation. This plausibly explains the experimentally observed significant reduction in AR protein levels, particularly for AR-FL.

After identifying upstream targets, we further elucidated midstream signaling events. ChIP-qPCR experiments confirmed AR's direct binding to the MBOAT2 gene promoter, establishing MBOAT2 as a key effector molecule downstream of the AR signaling pathway that regulates ferroptosis. MBOAT2 acts as a crucial barrier against ferroptosis by incorporating MUFA into phospholipids, thereby reducing the proportion of PUFA in membrane phospholipids and diminishing substrates for lipid peroxidation at the source [5, 40]. Our study demonstrates that Nid weakens AR's transcriptional activation of MBOAT2 by reducing AR protein levels, leading to downregulated MBOAT2 expression. This disruption unbalances intracellular lipid metabolism, causing PUFA-PL accumulation and increased susceptibility of lipid bilayers to peroxidation, ultimately driving cells toward ferroptosis.

Previous studies predominantly advocated that GPX4 serves as a core regulator in the classical ferroptosis pathway. However, research by Liang et al. revealed that the AR-MBOAT2 axis constitutes an independent ferroptosis-inducing pathway distinct from the GPX4 system, exhibiting particular significance in prostate cancer cells [29]. Our study shows that Nid induced ferroptosis primarily occurs through the HSP70-AR-MBOAT2 axis. The core mechanism we propose is as follows: Targeting HSP70 disrupts its interaction with the AR, thereby downregulating MBOAT2 expression. This disrupts lipid metabolism, triggering intense lipid peroxidation reactions that ultimately result in ferroptosis. To robustly support the conclusion that this specific axis induces ferroptosis, we present multi-level evidence in this study (Figs. 2, 3, 4). Within a research framework that is independent of GPX4, Nid induces ferroptosis via the HSP70-AR-MBOAT2 pathway.

The innovation of this study lies in the discovery of Nid, a novel small-molecule inhibitor of HSP70 that induces ferroptosis, providing a new chemical entity for drug development [41]. Furthermore, it established the association between HSP70 chaperone function and both androgen receptor transcriptional activity and ferroptosis susceptibility, revealing the upstream core role of protein homeostasis regulation in cell fate determination. Unfortunately, the quantity of Nid obtained from the fungus *Aspergillus* sp. SCSIO 41323 was insufficient. Our next step is to explore methods for large-scale production of Nid, followed by pharmacokinetic and pharmacodynamic studies in small and large animal models. Our study systematically elucidates the novel mechanism by which the small-molecule compound Nid targets the molecular chaperone HSP70 to destabilize the AR protein, thereby downregulating its downstream target gene MBOAT2 and ultimately inducing ferroptosis in prostate cancer cells. This discovery not only provides a novel therapeutic candidate for PCa but, more importantly, reveals a previously unrecognized signaling axis linking protein homeostasis regulation, androgen signaling pathways, and ferroptosis. It offers new theoretical foundations and therapeutic strategies for overcoming resistance to AR pathway inhibitors.

## Materials and methods

### Compound preparation

The fungus *Aspergillus* sp. SCSIO 41323 (preservation no. GDMCC 66152) was cultivated under static conditions for metabolite production. Seed medium containing malt extract (15 g/L) and sea salt (32 g/L) in distilled water (pH 6.5) was inoculated with the strain and incubated at 24 °C with shaking at 180 rpm for 3 days. Subsequently, the seed culture was transferred (5% v/v inoculation) into a solid-state fermentation medium of 60 × 1 L Erlenmeyer flasks consisting of rice (150 g/L) and sea salt (32 g/L) in distilled water. The fermentation was carried out statically at 24 °C for 30 days.

**Fig. 6:**
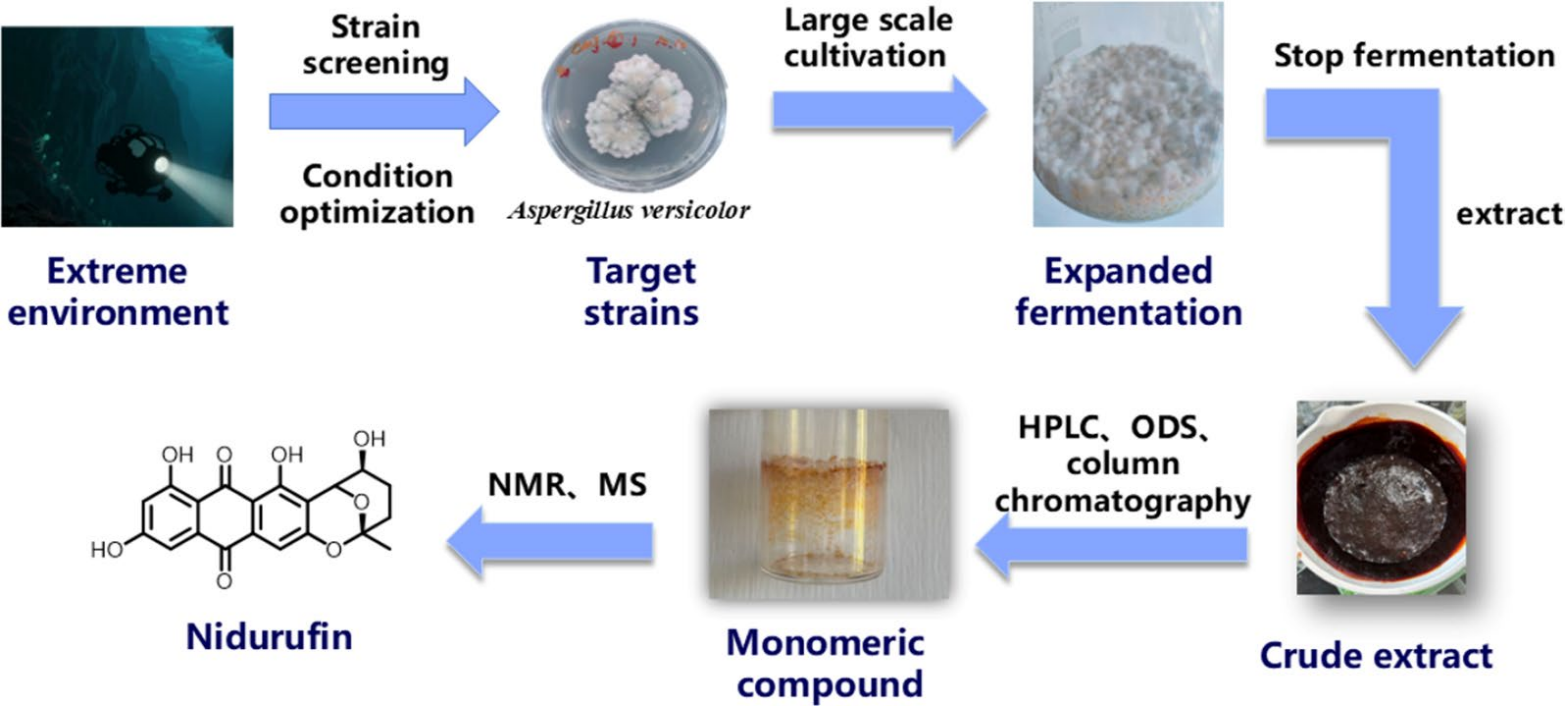
The extraction and separation process of Nid and its molecular structure

The fermented material was extracted twice with ethyl acetate. The combined organic layers were concentrated under reduced pressure to afford a crude extract (31.0 g). The extract was subjected to medium-pressure liquid chromatography (MPLC) on normal-phase silica gel, eluting with a gradient of dichloromethane/methanol (100:0 to 0:100, v/v), to yield eight fractions (Fr.1–Fr.8). Fr.2 (8.5 g), eluted with DCM/MeOH (100:1), was further separated by ODS reversed-phase column chromatography using a gradient of MeOH/H₂O (0:100 to 100:0), yielding nine subfractions (Fr.2-1–Fr.2-9). Subfraction Fr.2-7 (0.8 g), obtained with MeOH/H₂O (80:20–85:15), was purified by semi-preparative HPLC (YMC-pack ODS-A, 10 × 250 mm, 5 μm) with an isocratic elution of MeOH/H₂O (80:20) at a flow rate of 2.5 mL/min (detection at 220 nm), to afford eight subfractions (Fr.2-7-1–Fr.2-7-8). Fr.2-7-4 (retention time 19–20 min, 18.4 mg) was further purified by semi-preparative HPLC under isocratic conditions with CH_3_CN/H₂O (65:35) at 3 mL/min, yielding Nid (8.07 mg, t_R_ = 10 min, Purity: > 95%) (Fig. 6, Figure S3, S4, S5).

### Primary reagents, Antibodies

The primary reagents and antibodies used in this study are as follows: RPMI 1640 medium (Cat# C11875500BT), DMEM F12 medium (Cat# C11330500BT), DMEM medium (Cat# C11995500BT), MEM medium (Cat# 11090081), and Keratinocyte-SFM medium (Cat# 37000-015) were purchased from Gibco. Charcoal-Stripped FBS was purchased from Biological Industries (Cat# 04-201-1A), fetal bovine serum (FBS, Cat# F0193) and penicillin–streptomycin solution (100 × , Cat# 20200429) were purchased from Solarbio. MTT (Cat# M8180), crystal violet staining solution (Cat# G1063), dimethyl sulfoxide (cell culture grade, Cat# D8371), and dihydrotestosterone (DHT, Cat# ID0310) were purchased from Solarbio Life Science Co., Ltd. Dimethyl sulfoxide (DMSO, Cat# D103272) was also purchased from Aladdin. Docetaxel (DOC, Cat# S1148) and Enzalutamide (Cat# S1250) were purchased from Selleck. NuPAGE™ 10% Bis–Tris Gels (Cat# NP0302BOX, NP0303BOX), CellTracker™ CM-DiI Fluorescent Dye (Cat# C7000), TRIzol™ Reagent (Cat# 15596026CN), and RevertAid™ Master Mix Kit (Cat# M1631) were purchased from Thermo Fisher Scientific. ChIP-IT® Express Enzyme Kit (Cat# 53009), ChIP DNA Purification Kit (Cat# CN58002), and RNAII Antibody (Cat# 91152) were purchased from Active Motif. The Reactive Oxygen Species Detection Kit (Cat# R6033-A) was purchased from U-Landy. The Live/Dead Cell Staining Kit (Cat# PF00008) and primary antibodies for Western blot, immunofluorescence, and ChIP experiments, including AR (Cat# 22089-1-AP), HSP70 (Cat# 66183-1-Ig), MBOAT2 (Cat# 17905-1-AP), IgG (Cat# 30000-0-AP), and GAPDH (Cat# 10494-1-AP) were purchased from Proteintech Group. Primers used for ChIP-qPCR analyses (MBOAT2-ChIP F: 5′-GTAGGTTTGGACTGGCAGCA-3′, R: 5′-CGTAGCACCACGCATTACTC-3′) were synthesized by Sangon Biotech (Shanghai). All other routine chemical reagents were of analytical grade.

### Cell culture

All human prostate cancer cell lines (22Rv1, PC-3, LNCaP, VCaP, and DU145) and normal prostate cell lines (RWPE-1 and WPMY-1) were purchased from the Chinese Academy of Sciences Cell Bank/Stem Cell Bank (Shanghai, China). Cells were cultured in their respective recommended media: 22Rv1 and LNCaP cells in RPMI-1640 medium; PC-3 cells in DMEM-F12 medium; DU145 cells in MEM, and DMEM medium (Gibco, China) for VCaP, and WPMY-1 cells. All media except for RWPE-1 cells were supplemented with 10% fetal bovine serum (FBS; Sigma, South American origin) and 1% penicillin–streptomycin solution. RWPE-1 cells were cultured in keratinocyte serum-free medium (K-SFM; Gibco, China) containing recombinant human epidermal growth factor (rEGF), supplemented with 25 μg/mL bovine pituitary extract and 0.1% gentamicin/amphotericin solution. All cells were cultured in a humidified incubator at 37 °C with 5% CO₂. For androgen deprivation experiments, charcoal- stripped fetal bovine serum replaced conventional FBS. All cell lines were routinely tested for mycoplasma contamination and used within 20 passages post-thaw. Cells were passaged at 80–90% confluence using 0.25% trypsin–EDTA solution for digestion and separation.

### MTT cell viability assay

Cell viability was assessed using the MTT assay. Cells in the logarithmic growth phase were seeded into 96-well plates (5 × 10^3^ cells/well). After 24 h of attachment, fresh medium containing varying concentrations of Nid was added, and cells were cultured for an additional 72 h. MTT solution was added to each well. After incubation at 37 °C for 4 h, the supernatant was carefully aspirated. DMSO was added to dissolve the formed formazan crystals. The absorbance values at 570 nm were measured using a microplate reader (Sybergy H1, BioTek). The experiment was independently repeated three times. GraphPad Prism 8.0 software was used to calculate the half-maximal inhibitory concentration (IC₅₀).

### Plate clone formation assay

Well-growing 22Rv1 cells were seeded at a density of 1 × 10^3^ cells per well in a 12-well plate. After the cells adhered to the plate, solutions of Nid at different concentrations (2.5, 5, and 10 μM) were added. The cells were then incubated at 37 °C in a 5% CO₂ incubator for 7 days. The size of the clones was observed under a microscope. The cells were then fixed with 4% paraformaldehyde for 30 min, the fixative was discarded, and the cells were washed three times with PBS buffer. The cells were stained with 0.1% crystal violet (Cat# G1063, Solarbio life sciences) for 15 min, the dye was discarded, and the cells were washed three times with PBS buffer and air-dried. The colonies were photographed and analyzed using a colony counter (GelCount, Oxford Optronix). This experiment was independently repeated three times.

### 3D cell sphere analysis

Log-phase 22Rv1 cells were seeded at a density of 5 × 10^3^ cells per well in 200 μL of medium and plated into U-bottom 96-well plates (Cat# 174925, Thermo Fisher Scientific). The plates were then centrifuged sequentially at 1200 rpm and 1500 rpm for 5 min to promote cell aggregation and spheroid formation at the well bottom. After 24 h of culture at 37 °C and 5% CO₂ to stabilize spheroid formation, cells were treated for 72 h with media containing DMSO (0.1%, v/v), docetaxel (1 μM), and varying concentrations of Nid (5, 10, 20 μM). Following treatment, spheroids were stained in the dark for 4 h using a live/dead cell staining kit (Cat# PF00008, Proteintech). After three-time washes with PBS, images were captured using an inverted fluorescence microscope (Zeiss Axio Vert. A1, Germany) to assess spheroid morphology, size, and intracellular cell viability. The experiment was independently repeated three times.

### Transmission electron microscopy

22Rv1 cells were seeded at a density of 1 × 10⁶ cells/well in 100 mm culture dishes. After 48 h of culture, cells were treated with 20 μM Nid for 48 h. Cells were collected after trypsin digestion, sequentially fixed with 2.5% glutaraldehyde followed by 1% osmium tetroxide, dehydrated using an acetone gradient, and embedded in dehydrating agents and Epon-812 embedding medium. Ultra-thin sections prepared were double-stained with uranium acetate and lead citrate, then observed and imaged using a transmission electron microscope (JEM-1400FLASH, JEOL).

### Cellular reactive oxygen species assay

22Rv1 cells were seeded at a density of 5 × 10^5^ cells per well in a 6-well plate. After the cells had fully adhered, two blank control groups were set up using DMSO (0.1%, v/v), one positive control group, and three treatment groups were treated with a series of Nid solutions (5, 10, 20 μM) for 72 h. The positive control group cells were pretreated with Rosup reagent (100 μM) using the ROS Detection Kit (R6033-A, U-Elandy) and incubated at 37℃ for 1 h before cell collection. Except for one blank control group, all other groups were incubated with DCFH-DA reagent diluted 1:2000 at 37 °C in the dark for 30 min, followed by two washes with PBS buffer. The relative intensity of ROS in live cells was detected by flow cytometry (FongCyte™, Challen Bio).

### Western blot

22Rv1 cells in logarithmic growth phase were seeded at a density of 2 × 10⁶ cells/well in a 6-well plate and cultured at 37 °C with 5% CO₂ for 24 h until fully confluent. Cells were then treated with Nid at concentrations of 5, 10, and 20 μM for 48 h. After treatment, cells were harvested, lysed thoroughly with pre-prepared lysis buffer (RIPA:PMSF:phosphatase inhibitor = 100:1:1), and the supernatant was collected by centrifugation as the total protein extract. Protein concentration was determined using the BCA method (23227, Thermo scientific). Protein samples were treated with NuPAGE™ LDS Sample Buffer (4 × ; NP0007, Invitrogen) and NuPAGE™ Sample Reducing Agent (10 × ; NP0009, Invitrogen), then separated by electrophoresis on a 10% NuPAGE Bis–Tris gel (NP030C, Invitrogen). The gel was subsequently transferred to a polyvinylidene difluoride (PVDF) membrane (ISEQ00010, Immobilon-PSQ). Following transfer, the PVDF membrane was blocked at room temperature for 1 h with TBST containing 5% nonfat milk. Subsequently, the membrane was incubated overnight at 4 °C with the corresponding primary antibody, washed, and then incubated at room temperature for 1 h with HRP-labeled secondary antibody (1:10,000). Finally, chemiluminescence was used for signal detection, and images were captured using the ChemiDoc™ CD Touch imaging system (Bio-Rad, USA). Experiments were independently repeated at least three times. Further analysis and processing were performed using Image Lab Software 6.0 (Bio-Rad, China).

### Real-time quantitative PCR

Logarithmically growing, well-established 22Rv1 cells were digested, centrifuged, and counted. Cells were then seeded at a density of 1 × 10⁶ cells per well in a 6-well plate and cultured at 37 °C with 5% CO₂ for 24 h. Following cell attachment, the cells were treated with various concentrations of Nid (5 μM, 10 μM and 20 μM) and 20 μM of enzalutamide (ENZ) for 24 h, after which the RNA was extracted. Cells were collected and RNA was extracted using the Trizol™ Reagent Kit (15596026CN, Invitrogen) according to the manufacturer's protocol. Subsequently, RNA was reverse transcribed into cDNA using the RevertAid™ Master Mix Kit (M1631, Thermo Fisher). Finally, quantitative PCR amplification was performed according to the 2 × qPCR Master Mix instructions (22204, TOLOBIO) qPCR reactions were completed on a Roche LightCycler 96 instrument, with data analyzed using LightCycler 96 software. GAPDH served as the internal control for this experiment, with each sample independently replicated three times.

### Immunofluorescence assay

96-well black culture plates were pre-coated with 1 × polylysine. Log-phase 22Rv1 cells were trypsinized, resuspended by centrifugation, counted, and seeded at a density of 1 × 10^4^ cells per well into the coated 96-well plate. Cells were cultured at 37 °C in 5% CO₂ until confluent. Following adhesion, cells were treated with 10 μM Nid, 10 nM DHT, or a combination of DHT and Nid for 12 h. Following treatment, cells were fixed with 4% paraformaldehyde for 20 min, permeabilized with 0.5% Triton X-100 for 15 min, and blocked with 1% bovine serum albumin (BSA) for 1 h. Primary anti-AR antibody was added and incubated overnight at 4 °C. After washing with PBS, corresponding fluorescently labeled secondary antibodies were added and incubated at room temperature in the dark for 1 h. Cell nuclei were stained with Hoechst 33342 for 5 min. Following PBS washing, images were acquired using an inverted fluorescence microscope (Zeiss Axio Vert. A1, Germany) with a 20 × objective.

### Chromatin immunoprecipitation-quantitative PCR

Log-phase 22Rv1 cells were digested and centrifuged, then adjusted to a density of 5 × 10⁶ cells/dish and seeded into 150 mm culture dishes. Cells were cultured at 37 °C with 5% CO₂ until confluent. After treating cells with either 0.1% DMSO (solvent control) or 20 μM Nid for 48 h, chromatin immunoprecipitation was performed using the ChIP-IT® Express Enzymatic Kit (53009, Active Motif). The brief workflow included: cell fixation, chromatin fragmentation by enzymatic digestion, immunoprecipitation (using AR antibody and corresponding IgG control), chromatin elution, and deco valent binding release. Purified DNA was recovered using the ChIP DNA Purification Kit (CN58002, Active Motif). qPCR analysis assessed AR enrichment at target gene promoter regions, normalized against Input DNA as a reference. The primer sequences (MBOAT2-ChIP, F: 5′-GTAGGTTTGGACTGGCAGCA-3′, R: 5′-CGTAGCACCACGCATTACTC-3′) were derived from Liang’s article [29].Experiments were performed on the Roche LightCycler 96 system with three independent replicates per group. Data were analyzed using LightCycler 96 software.

### Molecular docking study

The crystal structure of HSP70 (PDB: 3I33) was obtained from the RCSB PDB database [42]. Molecular docking was performed using AMDock software (v1.5.2) and the CB-Dock2 website to simulate ligand-receptor interactions, optimize binding conformations, and predict binding sites. The software calculates intermolecular forces to refine spatial conformations and relative positioning, utilizing AMDock's automated docking box to predict optimal binding modes and potential binding sites. The docking results were then visualized using PyMOL to identify binding sites between the small molecule and the protein. To further elucidate binding details, the energy-optimized complex structure was submitted to the PLIP (Protein–Ligand Interaction Profiler) online platform for systematic analysis of key intermolecular interactions, including hydrogen bonds and hydrophobic interactions.

### Cellular thermal shift assay

Take 22Rv1 cells in good condition during the logarithmic growth phase. After digestion, centrifugation, and counting, adjust the cell density to 5 × 10⁶ cells per dish and seed into two 150 mm culture dishes. Incubate the cells at 37 °C in a 5% CO₂ incubator. After cell attachment, treat 22Rv1 cells with DMSO (0.1%) and Nid (20 μM) for 12 h. Following cell collection, resuspend the 22Rv1 cell pellet in 150 μL PBS buffer containing PMSF (PBS: PMSF = 100: 1) per tube. After sonication for 10 min, each cell suspension was equally distributed into 1.5 mL centrifuge tubes (30 μL per tube). Samples were frozen at -80 °C for 30 min, thawed at room temperature for 30 min, and subjected to three freeze–thaw cycles to lyse cells and obtain whole-cell extracts. Establish five temperature gradients at 52 °C, 55 °C, 58 °C, 61 °C, and 64 °C, heating each temperature for 3 min. After centrifugation, transfer the supernatant to a new centrifuge tube and determine protein concentration using the BCA Protein Quantification Kit (23227, ThermoFisher Scientific). Protein expression levels were detected using the Western blot method described above.

### Prostate cancer xenograft model in zebrafish

Wild-type AB strain zebrafish were provided by the Zebrafish Platform of the Zebrafish Laboratory of Guangxi Key Laboratory of Efficacy Study on Chinese Materia Medica, with rearing conditions following the standard protocols of the National Zebrafish Resource Center. All experimental protocols were approved by Guangxi University of Chinese Medicine Institutional Welfare and Ethical Committee (Approval No. DW20220525-090–09). Log-phase 22Rv1 cells were labeled with D-PBS containing 1 μg/mL CellTracker™ CM-DiI dye (C7000, Invitrogen) through incubation at 37 °C for 5 min followed by 15 min of dark-protected staining at 4 °C. After staining, cells were collected by centrifugation at 1000 rpm for 5 min, washed three times with D-PBS, and finally resuspended in 50 μL RPMI-1640 medium for subsequent use. Zebrafish were anesthetized with 0.1% MS-222 and fixed on 1.5% agarose gel. Under micromanipulation, 1–2 nL of cell suspension was injected into the yolk sac region. Successfully inoculated individuals (verified by fluorescence) were randomly assigned to the following groups (n = 8): 0.1% DMSO solvent control, 1 μM docetaxel group, and Nid (5, 10, 20 μM) experimental groups, with continuous treatment for 72 h. Daily observations of zebrafish survival were conducted throughout the treatment period. Following completion of drug administration, zebrafish were refixed in 1.5% agarose gel. Fluorescence microscopy imaging was performed, and ImageJ software was utilized to quantify fluorescence intensity in tumor regions to assess in vivo tumor growth inhibition effects.

### Statistical analysis

All statistical analyses were performed using GraphPad Prism 8.0 (GraphPad Software, USA). Experimental data are expressed as mean ± standard deviation (SD). Differences between groups were assessed using one-way analysis of variance (ANOVA), followed by Dunnett's T-test for multiple comparisons. Statistical significance was defined as ^*^P < 0.05 and ^**^P < 0.01.

## Supplementary Information

Additional file 1.

## Data Availability

The original contributions presented in the study are included in the article/Supplementary Material; further inquiries can be directed to the corresponding author.

## Abbreviations

PCa: Prostate cancer
AR: Androgen receptor
Nid: Nidurufin
HSP70: Heat Shock Protein 70
ADT: Androgen deprivation therapy
CRPC: Castration-resistant prostate cancer
AR-V: AR variants
GPX4: Glutathione peroxidase 4
MBOAT2: Membrane-associated O-acyltransferase domain protein 2
MUFAs: Monounsaturated fatty acids
HSP: Heat shock protein
MPLC: Medium-pressure liquid chromatography
DHT: Dihydrotestosterone
DMSO: Dimethyl sulfoxide
DOC: Docetaxel
FBS: Fetal bovine serum
K-SFM: Keratinocyte serum-free medium
rEGF: Recombinant human epidermal growth factor
PVDF: Polyvinylidene difluoride
BSA: Bovine serum albumin
CETSA: Cellular Thermal Shift Assay
SD: Standard deviation
ANOVA: Analysis of variance
TEM: Transmission electron microscopy
ROS: Reactive oxygen species
AR-FL: Full-length androgen receptor
ENZ: Enzalutamide
ChIP-qPCR: Chromatin immunoprecipitation-Quantitative polymerase chain reaction
