# Supplementary material for: Targeting Hsp70 triggers ferroptosis: a novel anti-cancer mechanism of a marine natural product in prostate cancer
Source: Nat Prod Bioprospect. 2026 Feb 5;16(1):34. doi: 10.1007/s13659-025-00586-9 (PMC12872954; doi:10.1007/s13659-025-00586-9)
Supplement: Supplementary file 1 — Additional file 1. [file 13659_2025_586_MOESM1_ESM.docx]

**Targeting Hsp70 Triggers Ferroptosis: A Novel Anti-Cancer Mechanism of a Marine Natural Product in Prostate Cancer**

Qiuyu Liu ^a, c, #^, Mengjing Cong ^b, #^, Chenghai Gao ^c^, Yonghong Liu ^b, c, *^, Junfeng Wang ^b, *^, Xueni Wang ^a, c, d *^

^a^ Guangxi Engineering Research Center for High-Value Utilization of Guangxi-Produced Authentic medicinal Herbs, Institute of Traditional Chinese and Zhuang-Yao Ethnic Medicine, Guangxi University of Chinese Medicine, Nanning 530200, China

^b^ State Key Laboratory of Tropical Oceanography/Guangdong Key Laboratory of Marine Materia Medica, South China Sea Institute of Oceanology, Chinese Academy of Sciences, Guangzhou 510301, China

^c^ Guangxi key laboratory of marine drugs, Institute of marine drugs, Guangxi University of Chinese Medicine, Nanning 530200, China

^d^ Guangxi Innovation Center of Zhuang Yao Medicine, Institute of Traditional Chinese and Zhuang-Yao Ethnic Medicine, Guangxi University of Chinese Medicine, Nanning 530200, China

^#^ These authors contributed equally to this work.

^*^ Corresponding author. E-mail: Yonghong Liu, yonghongliu@scsio.ac.cn; Junfeng Wang, [wangjunfeng@scsio.ac.cn](mailto:wangjunfeng@scsio.ac.cn); Xueni Wang, [wangxueni@gxtcmu.edu.cn](mailto:wangxueni@gxtcmu.edu.cn).

# Supplementary figure and table


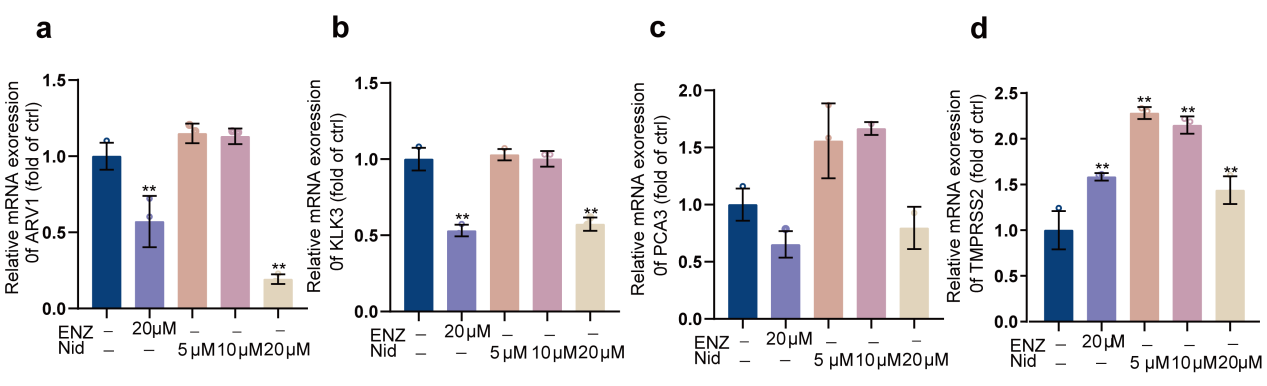
1. Figure S1 The regulatory role of Nid in the expression of ARV1 and AR target gene KLK3, PCA3, TMPRSS2 in 22Rv1 cells. 22Rv1 cells were treated with Nid (5, 10, 20 μM) and ENZ (20 μM) for 24 hours, respectively. (a) Changes in ARV1 mRNA expression. (b) Changes in KLK3 mRNA expression. (c) Changes in PCA3 mRNA expression. (d) Changes in TMPRSS2 mRNA expression.


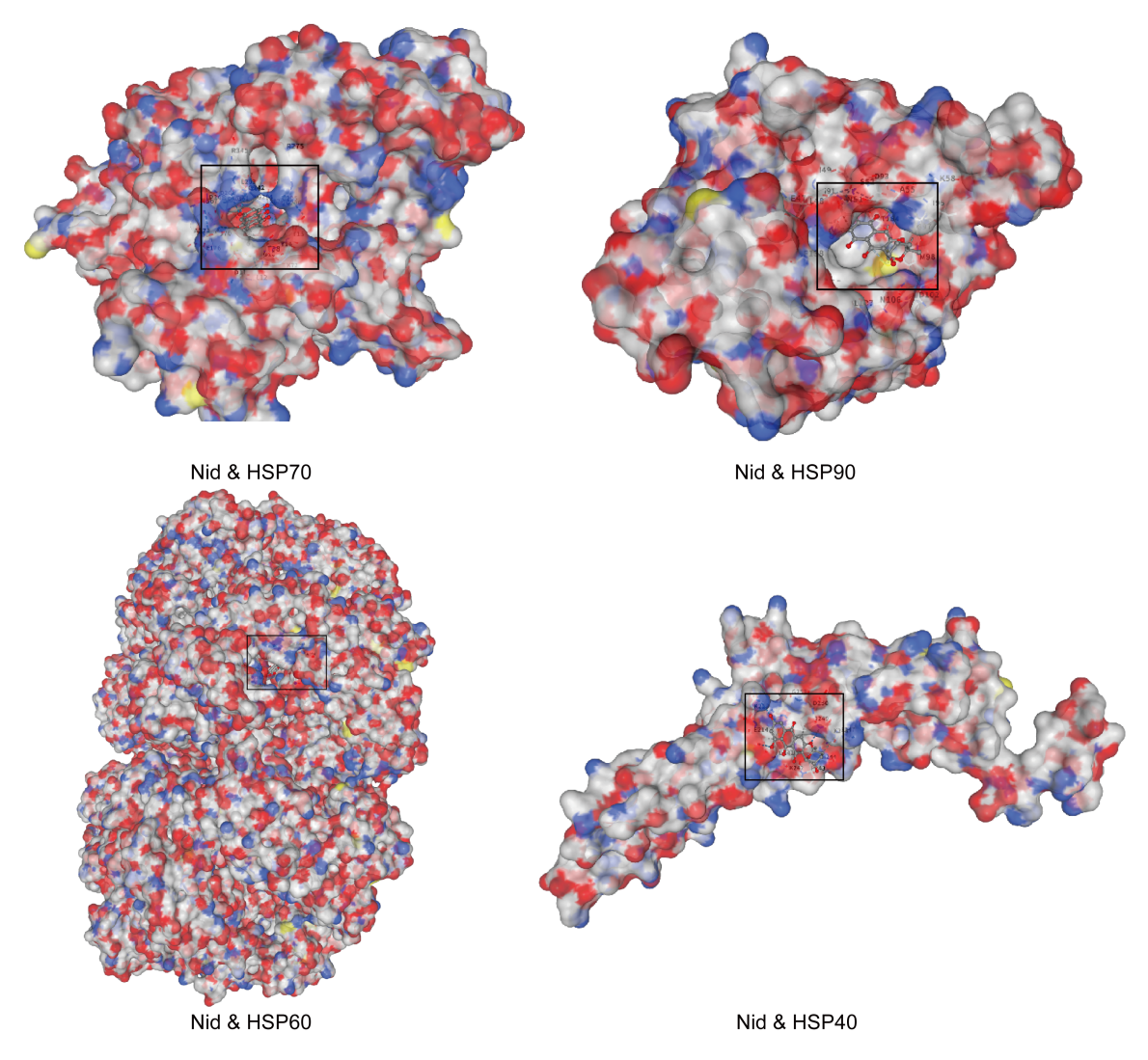


1. Figure S2 3D interaction pattern between Nid and Heat Shock Proteins. Molecular docking models of the small-molecule compound Nid with HSP70, HSP90, HSP60, and HSP40 proteins.

Figure S3. ^1^H NMR spectrum (500 MHz) of nidurufin in DMSO-*d*_6_.

Figure S4. ^13^C NMR spectrum (125 MHz) of nidurufin in DMSO-*d*_6_.

1. Figures S3-S4 represents the ^1^H, ^13^C NMR data of compound nidurufin.


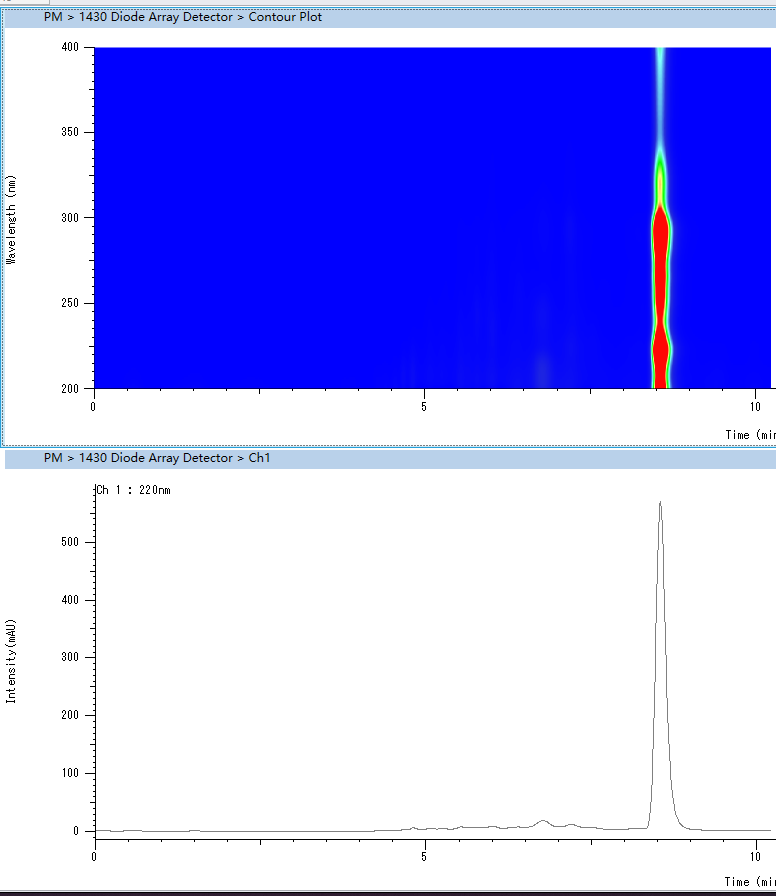


1. Figure S5 shows the HPLC analysis of the nidurufin isocratic elution of MeOH/H₂O (85:15) at a flow rate of 2.5 mL/min (detection at 220 nm).
2. Table S1 Molecular docking parameters for heat shock proteins and compound Nid.

| Protein Name | PDB ID | Vina score | Cavity volume (Å3) | Center (x, y, z) | Docking size (x, y, z) |
| --- | --- | --- | --- | --- | --- |
| HSP70 | 3I33 | -11.1 | 7898 | -10, -8, 8 | 32, 35, 22 |
| HSP90 | 1BYQ | -9 | 705 | 40, -48, 65 | 22, 22, 22 |
| HSP60 | 7AZP | -7.6 | 1379 | 97, 176, 136 | 22, 22, 22 |
| HSP40 | 2QLD | -7 | 63 | 65, 71, 12 | 22, 22, 22 |

Import the Nid molecule into the CB-Dock2 website and perform molecular docking with heat shock proteins to obtain the optimal binding poses, in order to determine the best docking sites and various interactions. (Vina scores < -6 indicate moderate binding affinity, while scores < -9 indicate excellent affinity; lower scores indicate stronger binding.)

# Uncropped blot images

1. Fig.3a in our manuscript represents the analysis outcomes of the whole uncropped images in Sup.M.Fig.1.


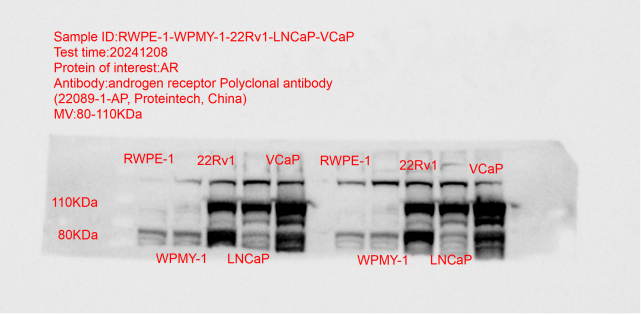

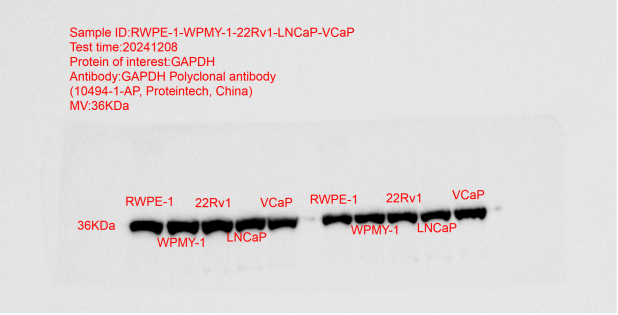


Sup.M.Fig.1 Original western blots images of AR in RWPE-1, WPMY-1, 22Rv1, LNCaP, and VCaP cells

1. Fig.3c in our manuscript represents the analysis outcomes of the whole uncropped images in Sup.M.Fig.2.


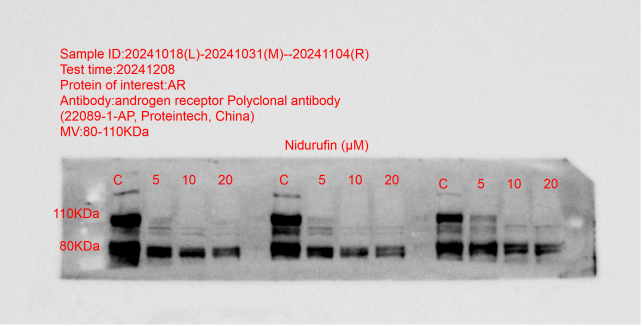

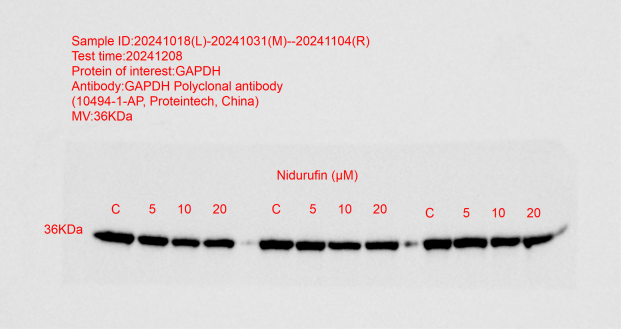


Sup.M.Fig.2 Original western blots images of AR in 22Rv1 cells.

1. Fig.4a in our manuscript represents the analysis outcomes of the whole uncropped images in Sup.M.Fig.3.


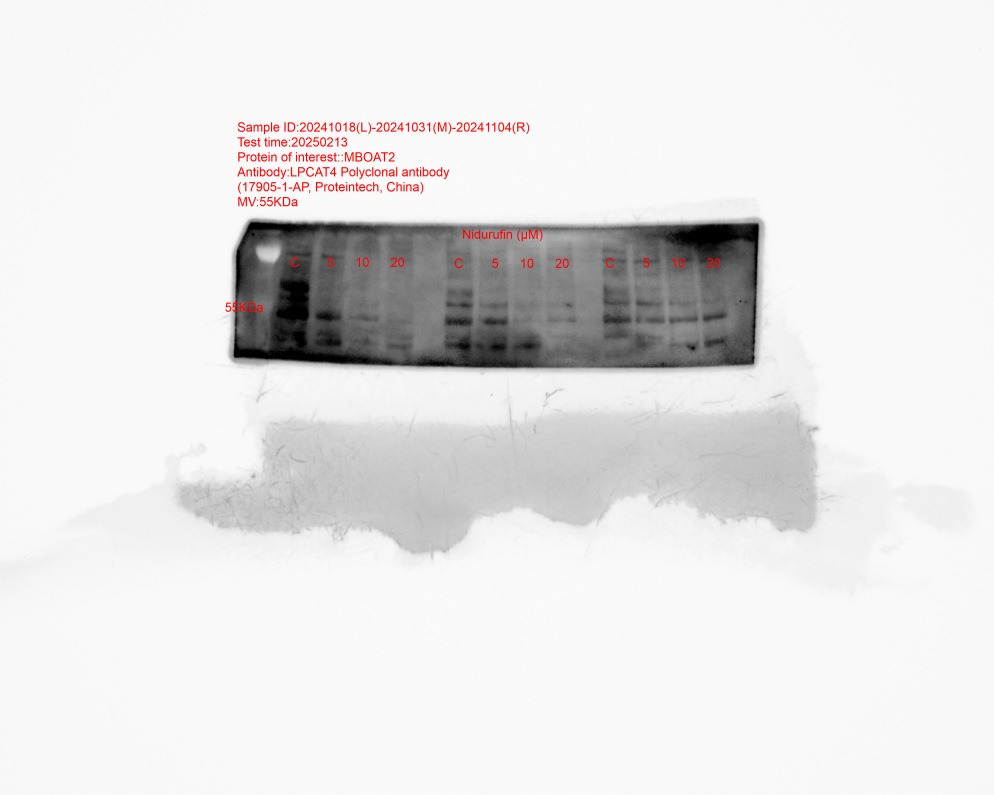

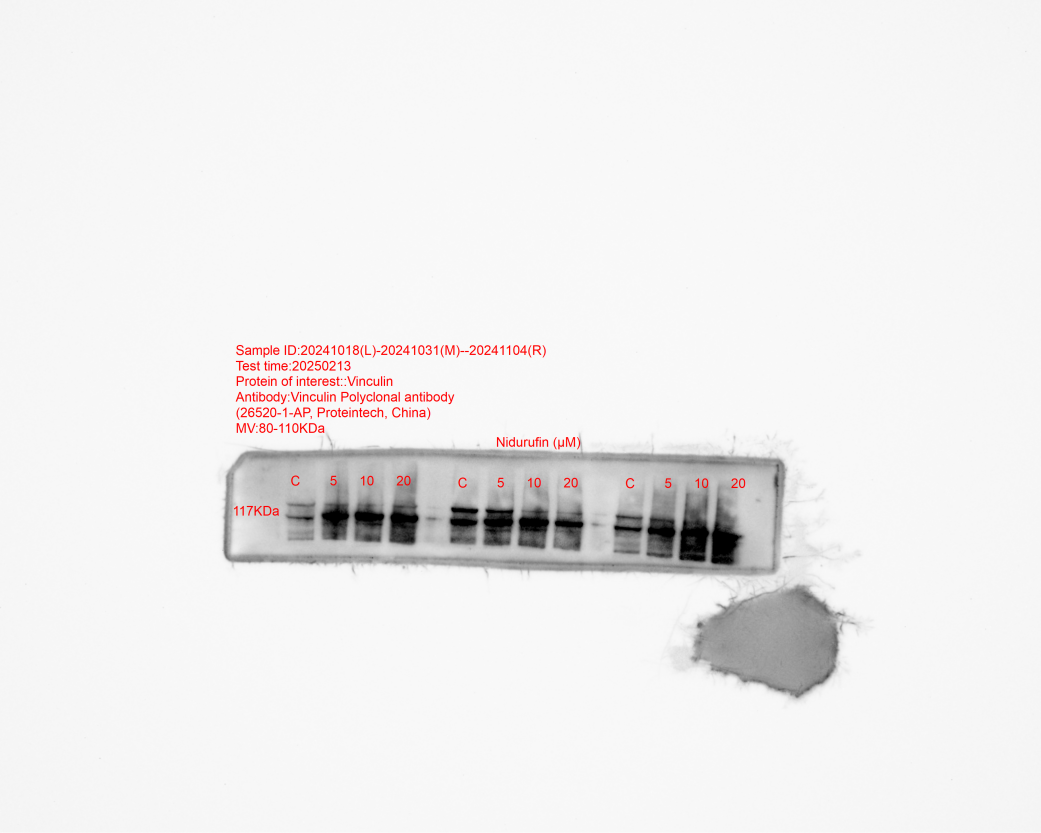


Sup.M.Fig.3 Original western blots images of MBOAT2 in 22Rv1 cells.

1. Fig.4f in our manuscript represents the analysis outcomes of the whole uncropped images in Sup.M.Fig.4.


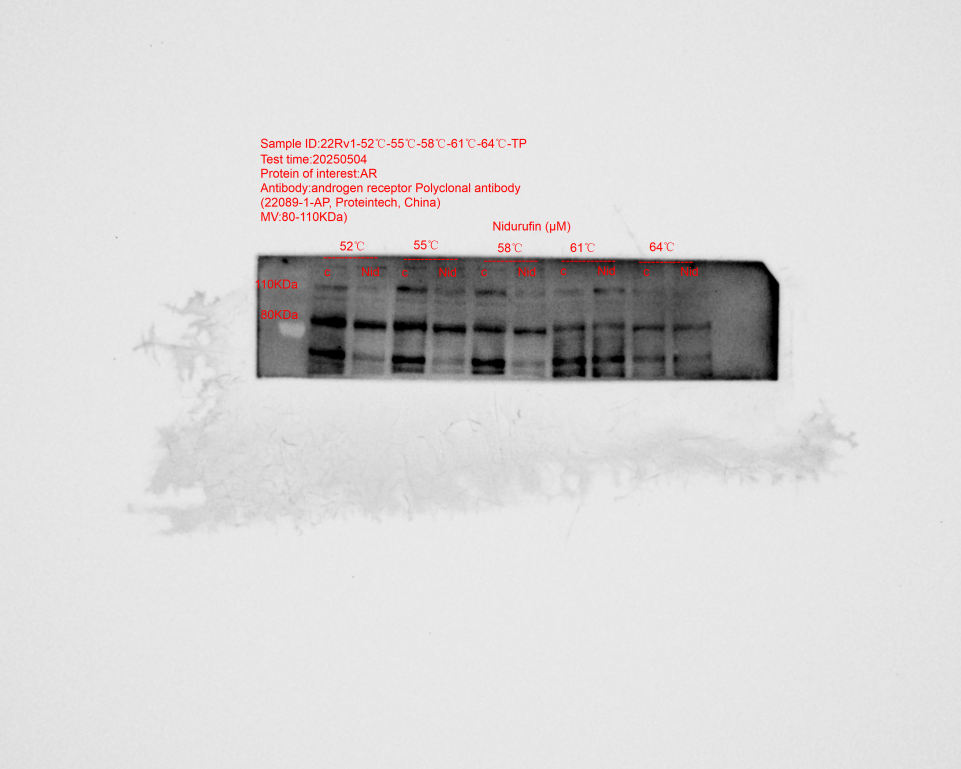

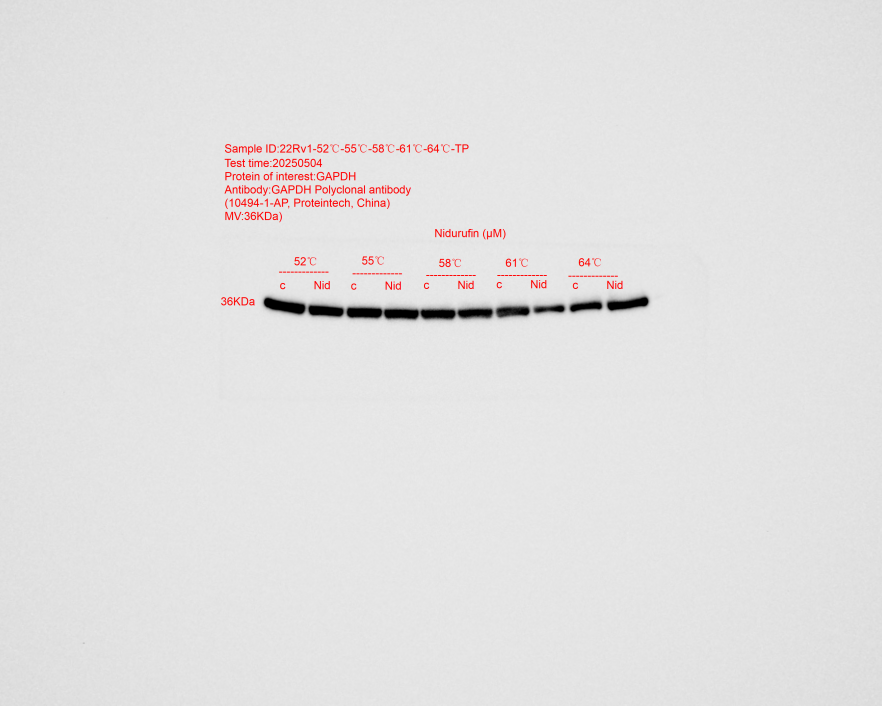


Sup.M.Fig.4 Original western blots images of AR in 22Rv1 cells.

1. Fig.4g in our manuscript represents the analysis outcomes of the whole uncropped images in Sup.M.Fig.5.


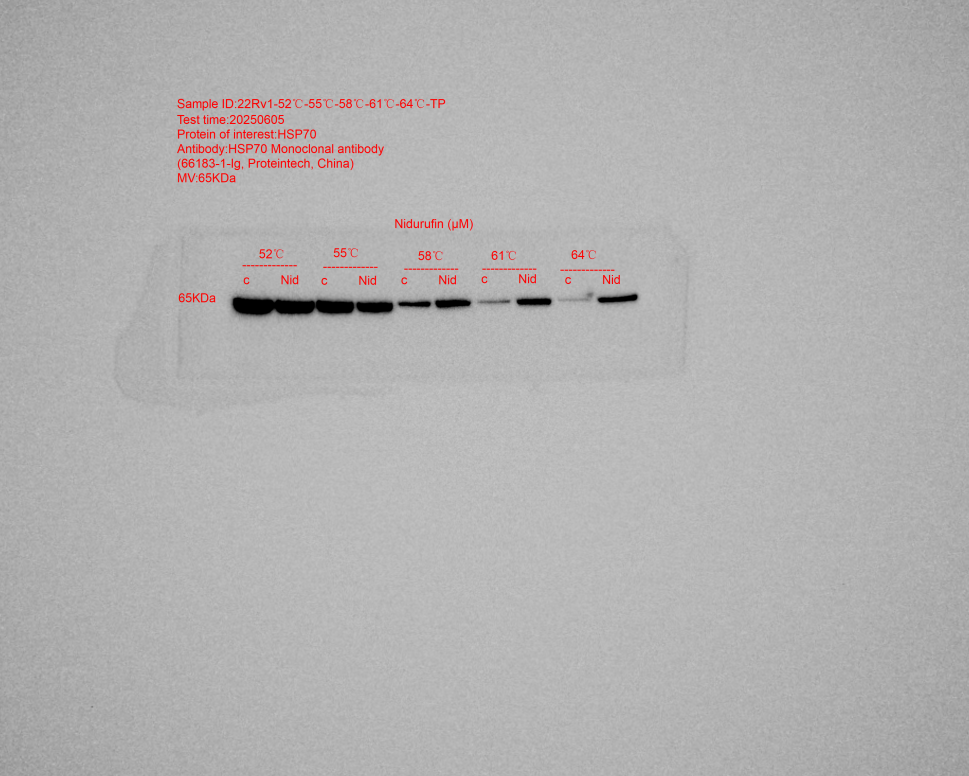

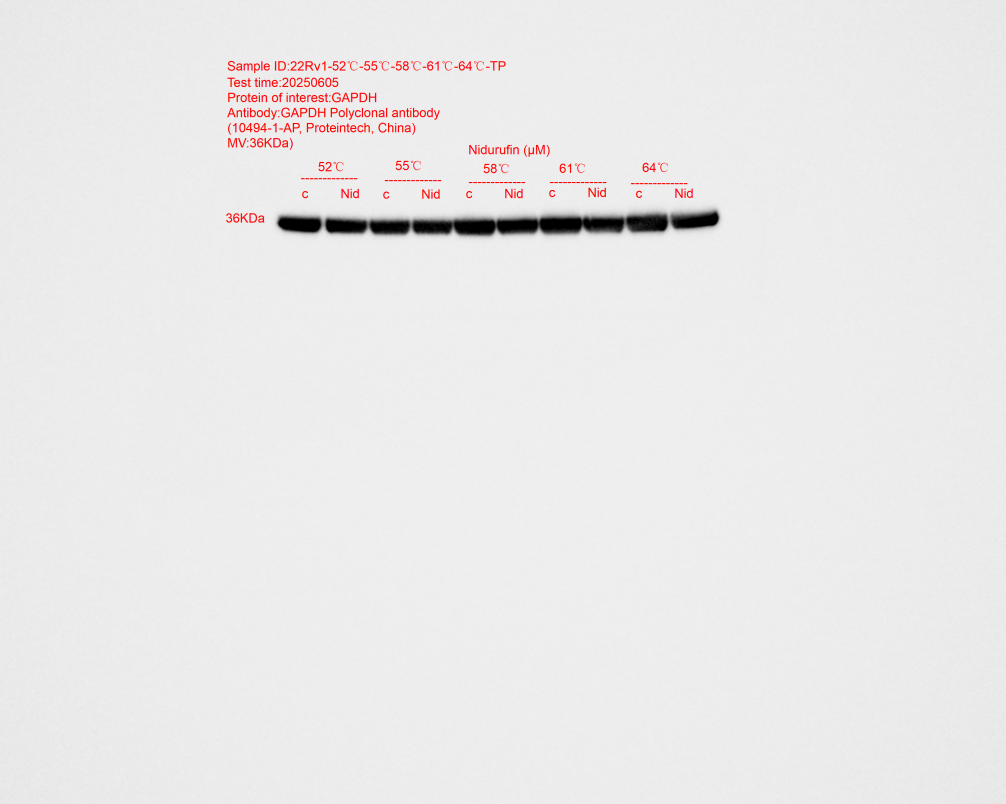


Sup.M.Fig.5 Original western blots images of HSP70 in 22Rv1 cells.
